# A gel-based PCR method to differentiate sheeppox virus field isolates from vaccine strains

**DOI:** 10.1186/s12985-018-0969-8

**Published:** 2018-04-02

**Authors:** Tesfaye Rufael Chibssa, Reingard Grabherr, Angelika Loitsch, Tirumala Bharani K. Settypalli, Eeva Tuppurainen, Nick Nwankpa, Karim Tounkara, Hafsa Madani, Amel Omani, Mariane Diop, Giovanni Cattoli, Adama Diallo, Charles Euloge Lamien

**Affiliations:** 1 Animal Production and Health Laboratory, Joint FAO/IAEA Agricultural and Biotechnology laboratory, Division of Nuclear Techniques in Food and Agriculture, Department of Nuclear Sciences and Applications, International Atomic Energy Agency, Wagramer Strasse 5, P.O. Box 100, A1400 Vienna, Austria; 2 Institute of Biotechnology, University of Natural Resources and Life Sciences (BOKU), Muthgasse 18, 1190 Vienna, Austria; 3 National Animal Health Diagnostic and Investigation Center (NAHDIC), P.O Box, 04, Sebeta, Ethiopia; 4 Institute for Veterinary Disease Control, Austrian Agency for Health and Food Safety (AGES), Modling, Austria; 5 Capripoxvirus Reference Laboratory, Pirbright Institute, Pirbright, Surrey, UK; 6 Pan African Veterinary Vaccine Centre, African Union (PANVAC), P. O. Box 1746, Debre Ziet, Ethiopia; 7 Institut National de la Médecine Vétérinaire, Laboratoire Central Vétérinaire d’Alger, Algiers, Algeria; 8 Laboratoire National de l’Elevage et de Recherches Vétérinaires, Institut Sénégalais de Recherches Agricoles Route du Front de Terre Hann, Dakar, Sénégal; 9 UMR CIRAD-INRA, Animal, Santé, Territoires, Risques et Ecosystèmes (ASTRE), cedex, 05 Montpellier, France

**Keywords:** CaPV, Sheeppox vaccine, Sheeppox virus, VARV B22R homologue gene, DNA ligase gene

## Abstract

**Background:**

Sheeppox (SPP) and goatpox (GTP) caused by sheeppox virus (SPPV) and goatpox virus (GTPV), respectively of the genus *Capripoxvirus* in the family *Poxviridae*, are severely afflicting small ruminants’ production systems in Africa and Asia. In endemic areas, SPP and GTP are controlled using vaccination with live attenuated vaccines derived from SPPV, GTPV or Lumpy skin disease virus (LSDV).

Sometimes outbreaks occur following vaccination. In order to successfully control the spread of the virus, it is essential to identify whether the animals were infected by the field strain and the vaccine did not provide sufficient protection. Alternatively, in some cases the vaccine strain may cause adverse reactions in vaccinated animals or in rare occasions, re-gain virulence. Thus, diagnostic tools for differentiation of virulent strains from attenuated vaccine strains of the virus are needed.

The aim of this study was to identify an appropriate diagnostic target region in the capripoxvirus genome by comparing the genomic sequences of SPPV field isolates with those of the most widely used SPP vaccine strains.

**Results:**

A unique 84 base pair nucleotide deletion located between the DNA ligase gene and the VARV B22R homologue gene was found only in SPPV vaccines derived from the Romanian and Yugoslavian RM/65 strains and absent in SPPV field isolates originated from various geographical locations of Asia and Africa.

In addition, we developed and evaluated a conventional PCR assay, exploiting the targeted intergenic region to differentiate SPPV vaccine virus from field isolates. The assay produced an amplicon size of 218 bp for the vaccine strains, while the SPPV field isolates resulted in a 302 bp PCR fragment. The assay showed good sensitivity and specificity, and the results were in full agreement with the sequencing data of the PCR amplicons.

**Conclusion:**

The developed assay is an improvement of currently existing diagnostic tools and, when combined with a capripox virus species-specific assay, will enhance SPP and GTP diagnosis and surveillance and facilitate epidemiological investigations in countries using live attenuated SPP vaccines. In addition, for laboratories with limited resources, the assay provides a simple and cost-effective alternative for sequencing.

**Electronic supplementary material:**

The online version of this article (10.1186/s12985-018-0969-8) contains supplementary material, which is available to authorized users.

## Background

Sheeppox (SPP) and goatpox (GTP) are caused by sheeppox virus (SPPV) and goatpox virus (GTPV) of the genus *Capripoxvirus* of the family *Poxviridae* [1]. In endemic areas SPP and GTP have a serious economic impact on small ruminant production systems, causing losses in productivity, mortality, damaging skins and hides, as well as inflicting international trade restrictions [2]. They are listed in the group of economically important animal diseases for which outbreaks have to be notified immediately to the World Organization for Animal Health [3].

The main mode of virus transmission is the direct contact between diseased and non-infected animals, but indirect transmission may also occur [4]. Clinical signs of SPPV and GTPV infections are characterized by ocular and nasal discharge and pock-like lesions in the skin and mucosae of the respiratory and gastrointestinal tracts [2, 4, 5]. Most of the isolates are host specific and cause disease mainly in sheep or in goats, whereas some isolates can cause serious disease in both animal species [6].

SPP and GTP are endemic in many African, Middle Eastern and Asian countries and recurrent epidemics have occurred in Greece and Bulgaria in 2013–2014 [7] and in Greece in 2016 and 2017. SPPV is also circulating in the Russian Federation where it causes sporadic outbreaks of disease.

In endemic regions, control of the disease is through effective immunization using killed or live attenuated vaccines derived from SPPV, GTPV or Lumpy skin disease virus (LSDV). In general, live attenuated vaccines are the better choice as compared to inactivated vaccines, as they confer long-lasting immunity. For instance, the Yugoslavian RM65, the Romanian Fanar and KSGP0240 strains, the most commonly used vaccines strains against SPPV, are reported to provide high levels of protection [7]. The Yugoslavian RM65 is widely used in the Middle East, Asia and in the Horn of Africa, while the Romanian Fanar is used in India and Maghreb countries. The Yugoslavian RM65 was attenuated by 30 serial passages on ovine kidney cells, and the Romanian Fanar by 26 serial passages on lamb testis cells [7].

The KSGP 0240 is widely used in several endemic regions in Africa. Nevertheless, KSGP0240 has been shown, by sequencing, to be a LSDV, thus, it can be differentiated from virulent isolates of SPPV using available capripoxvirus genotyping methods [7].

However, when using live attenuated vaccines, the epidemiological investigation of outbreaks can become quite challenging. When outbreaks occur following vaccination, it is essential to identify whether the animals were infected by the field strain because the vaccine did not provide sufficient protection. Alternatively, in some cases the vaccine strain may cause adverse reactions in vaccinated animals or, in rare occasions, re-gain the virulence as suggested by Lee and co-workers for herpesvirus vaccine [8].

Unfortunately, the current live attenuated capripox (CaP) vaccines do not offer the possibility to differentiate vaccinated animals from infected ones. This creates a need to identify a suitable genomic target and develop molecular tools that would enable the differentiation of SPPV field isolates from vaccine strains to rule out the involvement of SPP vaccines during a CaP outbreak in a vaccinated herd. Such a tool will facilitate the management and control of CaP infections in small ruminants.

The present study describes the use of a suitable diagnostic target of the SPPV genome to develop an assay that can discriminate SPP vaccines derived from the Romanian and the Yugoslavian RM/65 strains from SPPV field isolates, to facilitate the diagnosis and surveillance of capripox virus (CaPV) infections in small ruminants.

## Methods

### Virus and nucleic acid extraction

The information related to the field isolates and vaccine strains of SPPV as well as other CaPVs used in this study are presented in Table 1. Viral multiplication was performed on embryonic skin cell from sheep (ESH-L cells) grown in Hank’s Minimum Essential Medium (MEM) supplemented with 10% foetal calf serum and 1% antibiotics. DNA was extracted from infected cell culture supernatants and clinical samples using the AllPrep DNA/RNA extraction kit (QIAGEN) following the manufacturer’s instructions. Extracted DNA was eluted with 80 μl elution buffer and stored at − 20 °C until further use.

**Table 1:** List of capripoxvirus isolates used in this study

Sample No.	Isolate/Strain name	Source	Sample type	Country	Collection-date	Host
1	SPPV Morocco vaccine	Biopharma/Morocco	Cell culture	Morocco	Unknown	Sheep
2	SPPV Algeria vaccine Lot/7	INMV-LCV/Algeria	Cell culture	Algeria	Unknown	Sheep
3	SPPV Senegal vaccine	LNERV-ISRA/Senegal	Cell culture	Iran	1966	Sheep
4	SPPV PANVAC/6 vaccine	PANVAC/Ethiopia	Cell culture	Kenya	2010	Sheep
5	SPPV Turkey/98 Corum	VCRI-Pendik/Turkey	Cell culture	Turkey	1998	Sheep
6	SPPV Oman/84	IAH-Pirbright/UK	Cell culture	Oman	1984	Sheep
7	SPPV Turkey/98 Denizli	VCRI-Pendik/Turkey	Cell culture	Turkey	1998	Sheep
8	SPPV Algeria/93 Djelfa	INMV-LCV/Algeria	Cell culture	Algeria	1993	Sheep
9	SPPV Algeria/05 Illizi	INMV-LCV/Algeria	Cell culture	Algeria	2005	Sheep
10	SPPV Turkey/98 Sivas	VCRI-Pendik/Turkey	Cell culture	Turkey	1998	Sheep
11	SPPV MOG/SP/T/2/07	IVM/Mongolia	Skin scrapping	Mongolia	2007	Sheep
12	SPPV MOG/SP/T/3/07	IVM/Mongolia	Skin scrapping	Mongolia	2007	Sheep
13	GTPV Iraq/61 Gorgan	IAH-Pirbright/UK	Cell culture	Iraq	1961	Goat
14	GTPV MOG/GP/T/6/08	IVM/Mongolia	Skin scrapping	Mongolia	2008	Goat
15	GTPV MOG/GP/L/5/08	IVM/Mongolia	Skin scrapping	Mongolia	2008	Goat
16	GTPV Awi/O13/2011	NAHDIC/Ethiopia	Skin scrapping	Ethiopia	2011	Goat
17	GTPV Bale/O14/2007	NAHDIC/Ethiopia	Skin scrapping	Ethiopia	2007	Goat
18	GTPV Giner/O15/2007	NAHDIC/Ethiopia	Skin scrapping	Ethiopia	2007	Goat
19	LSDV KS-1	HSL-AGES/Austria	Cell culture	Kenya	1976	Sheep
20	LSDV Egypt/89 Ismalia	HSL-AGES/Austria	Cell culture	Egypt	1989	Cattle
21	LSDV Guder/B5/2008	NAHDIC/Ethiopia	Skin scrapping	Ethiopia	2008	Cattle
22	LSDV Humbo/B23/2010	NAHDIC/Ethiopia	Skin scrapping	Ethiopia	2010	Cattle
23	SPPV Algeria vaccine Lot/10	INMV-LCV/Algeria	Cell culture	Algeria	Unknown	Sheep
24	SPPV Mauritania/85 Gorgol	LNERV-ISRA/Senegal	Cell culture	Mauritania	1985	Sheep
25	SPPV Turkey/98 Darica	VCRI-Pendik/Turkey	Cell culture	Turkey	1998	Sheep
26	SPPV MOG/SP/T/1/2006	IVM/Mongolia	Skin scrapping	Mongolia	2006	Sheep
27	GTPV Saudi Arabia/93	IAH-Pirbright/UK	Cell culture	Saudi Arabia	1993	Goat
28	SPPV Nigeria/77	IAH-Pirbright/UK	Cell culture	Nigeria	1993	Sheep
29	GTPV Turkey/98 Denizli	VCRI-Pendik/Turkey	Cell culture	Turkey	1998	Goat
30	GTPV Oman/84	IAH-Pirbright/UK	Cell culture	Oman	1984	Goat
31	GTPV MOG/GP/T/4/08	IVM/Mongolia	Skin scrapping	Mongolia	2008	Goat
32	GTPV Yemen/83	IAH-Pirbright/UK	Cell culture	Yemen	1983	Goat
33	GTPV Towele/O17/2013	NAHDIC/Ethiopia	Skin scrapping	Ethiopia	2013	Goat
34	GTPV Halasya/G18/2013	NAHDIC/Ethiopia	Skin scrapping	Ethiopia	2013	Goat
35	LSDV Galesa/B12/2008	NAHDIC/Ethiopia	Skin scrapping	Ethiopia	2008	Cattle
36	LSDV Sodo/B24/2010	NAHDIC/Ethiopia	Skin scrapping	Ethiopia	2010	Cattle
37	LSDV Chilimo/B11/2008	NAHDIC/Ethiopia	Skin scrapping	Ethiopia	2008	Cattle
38	LSDV Ambo/B8/2008	NAHDIC/Ethiopia	Skin scrapping	Ethiopia	2008	Cattle
39	LSDV Toke/B6/2008	NAHDIC/Ethiopia	Skin scrapping	Ethiopia	2008	Cattle
40	LSDV Ginchi/B10/2008	NAHDIC/Ethiopia	Skin scrapping	Ethiopia	2008	Cattle
41	LSDV Sodo/B22/2010	NAHDIC/Ethiopia	Skin scrapping	Ethiopia	2010	Cattle
42	LSDV Adama/B4/2011	NAHDIC/Ethiopia	Skin scrapping	Ethiopia	2011	Cattle
43	LSDV Ziway/B3/2011	NAHDIC/Ethiopia	Skin scrapping	Ethiopia	2011	Cattle
44	LSDV Asella/B2/2011	NAHDIC/Ethiopia	Skin scrapping	Ethiopia	2011	Cattle
45	LSDV Arsi/B1/2011	NAHDIC/Ethiopia	Skin scrapping	Ethiopia	2011	Cattle
46	LSDV Sundus/2012	CVRL/Sudan	Skin scrapping	Sudan	2012	Cattle

*Abbreviations*: *VCRI* Veterinary Control and Research Institute, *LNERV-ISRA* Laboratoire National de l’Elevage et de Recherches Vétérinaires, Institut Sénégalais de Recherches Agricoles, *INMV-LCV* Institut National de la Médecine Vétérinaire, Laboratoire Central Vétérinaire, *IVM* Institute of Veterinary Medicine, *IAH* Institute for Animal Health, *PANVAC* Pan African Veterinary Vaccine Centre, *NAHDIC* National Animal Health Diagnostic and Investigation Center, *HSL-AGES* High Security Laboratory, Austrian Agency for Health and Food Safety, *CVRL* Central Veterinary Research Laboratories

### Primer design and PCR

The target region was selected based on the alignment of the full genomes of three SPPVs, Sheeppox virus 10,700–99 (AY077832), Sheeppox virus A (AY077833) and Sheeppox virus NISKHI, (AY077834), retrieved from GenBank, and the unpublished genome of the Romanian vaccine strain used in Morocco. PCR primers, flanking a unique nucleotide deletion in the Romanian vaccine strain, were designed using Primer3Plus online tool. The target was an intergenic region located between the DNA ligase gene and the VARV B22R homologue gene of CaPVs corresponding to position 121,500–122,799 of SPPV A (AY077833). The primers (Table 2) were designed to amplify amplicons of 302 bp in SPPV field isolates and 218 bp fragments for SPPV vaccines. The specificity of the primers was checked by using the Basic Local Alignment Search Tool (BLAST). The primers were synthesized and purified by HPLC by Eurofins Genomics (Austria).

**Table 2:** Primers used in this study. The length of the predicted amplicons are given

Primer name	Sequences	Length	Amplicon size
SPPV_DIV_Fow	5’-ATCTGCTACAAGTTTTAACGAACTTA- 3’	26	218 bp (SPPV vaccines)
SPPV_DIV_Rev	5’-TGAATGTGATCTCATATCCTTATTG-3’	25	302 (SPPV field isolates and GTPV) and 336 (LSDV)

PCR was conducted in a reaction volume of 20 μl, containing 500 nM of each forward and reverse primers, 0.2 mM dNTPs, 2.5 U Taq DNA polymerase (QIAGEN), 1× PCR buffer, and 2 μl template DNA. The cycling conditions were as follow: 95 °C for 4 min followed by 35 cycles of 95 °C for 30 s, 58 °C for 30 s, and 72 °C for 30 s and a final extension at 72 °C for 2 min. PCR products were cheeked by electrophoresis on a 2% agarose gel for 1 h at 100 V.

### Preparation of controls

The PCR amplicons of SPPV Denizli and SPPV Morocco vaccine (Romanian vaccine strain) were selected to prepare positive control plasmids, representing the field isolates and vaccine strains respectively. For plasmid preparation, each amplicon was purified and ligated into pGEM-T Easy Vector Systems (Promega). The ligated products were used to transform DH5α competent cells (Invitrogen). Plasmids containing the inserts were purified from the positive bacteria clones using the PureYield Plasmid Midiprep System (Promega) following the manufacturer’s instructions. The purified plasmids were sequenced to confirm the presence of the correct target region and quantified using the Quant-iTPicoGreen dsDNA Assay Kit (Invitrogen) on a NanoDrop 3300 fluorospectrometer. The concentration of the plasmids was determined following the steps described by Lamien et al., (2011) and the plasmids were kept at − 20 °C until analysis.

### Analytical sensitivity and specificity of the assay

The analytical sensitivity of the method was assessed by amplifying 10-fold serial dilutions, from10^7^ to 100 copies/reaction, then follow by 80, 60, 40, 20, 10 and 1 copies/reaction dilutions, of plasmids containing the corresponding target of SPPV Denizli and SPPV Morocco vaccine. The lowest number of viral genome copies that could be detected by the assay was recorded.

The specificity was evaluated by amplifying DNA extracted from all available SPP vaccines and field isolates as well as GTPV and LSDV isolates from various geographical regions (Table 1). The genotype of each sample was confirmed using a capripox species specific PCR (Gelaye et al., 2013). Additionally, the specificity in amplifying only CaPVs was determined by attempting to amplify DNA extracted from Orf virus (ORFV), *Mycoplasma capricolum* ssp. *capripneumonia* (Mccp), Bovine herpes virus (BOHV), Bovine popular stomatitis virus (BPSV) and cDNA derived from peste des petits ruminants (PPR) virus (Additional file 1: Table S1).

### Sequence analysis

For all CaPVs used in this study, the targeted region of the genome was sequenced to confirm the accuracy of the assay. Thus, all amplified PCR products of the above described PCR reactions were purified using Wizard SV Gel and PCR Clean Up System (Promega) and sequenced commercially by LGC Genomics (Germany). The sequence data were edited and the fragments assembled using Vector NTI 11.5 software (Invitrogen). Multiple sequence alignments were performed using the CLUSTALW algorithm implemented in BioEdit 7.5 software package to compare SPPV field isolates and vaccine strains. All sequences were deposited in GenBank under accession number MG764242 to MG764286.

## Results

### Assay design and optimization

**Fig. 1:**
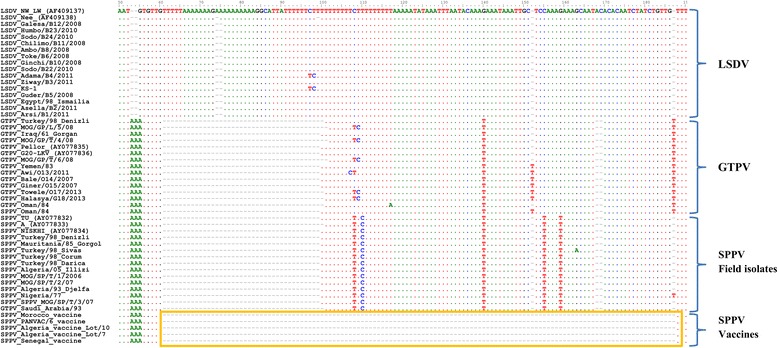
Multiple sequence alignments showing a 84-nucleotide deletion in SPPV vaccines. The nucleotide sequences of the intergenic region located between the DNA ligase gene and the VARV B22R homologue gene for 45 Capripoxviruses of this study and 7 other retrieved from Genbank were compared

Primers were designed to amplify a region between the DNA ligase gene and the VARV B22R homologue gene of SPPV. The region was first selected based on the sequence alignment of three SPPV publicly available full genome sequences and a draft genome sequence of the SPP Morocco vaccine derived from the Romanian vaccine strain. The SPP vaccine had an 84 bp nucleotide deletion as compared to SPPV field isolates (Fig. 1). The initial evaluation of the assay showed that the Morocco vaccine strain could be differentiated from SPPV Denizli field isolate based on the size difference of the produced PCR amplicons: 218 bp for the vaccine strain and 302 bp for the SPPV field isolate. In the subsequent steps, the assay was optimized and further evaluated.

### Evaluation of the assay

**Fig. 2:**
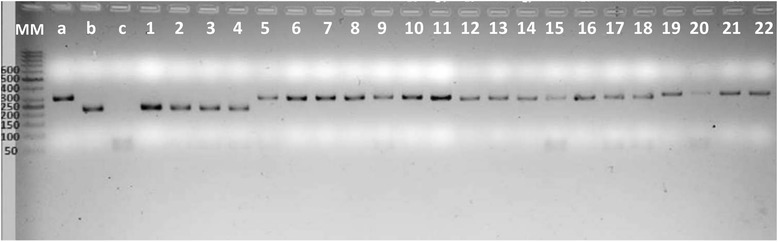
Gel picture of PCR products for selected capripoxvirus samples. The SPPV vaccines appear to be shorter than SPPV field isolates, GTPV and LSDV due to the 84 bp sequence difference. The PCR products of 218 bp, 302 bp and 338 bp represent SPPV vaccine strains, SPPV field isolates/GTPVs, and LSDVs respectively. MM: 50 bp DNA ladder; a: positive control plasmid of the SPPV field isolates; b: positive control plasmid of the SPPV vaccine strain; c: Negative control; Lanes 4–7: SPPV vaccine strains (sample 1 to 4 of Table 1); Lanes 8–15: SPPV field isolates (sample 5 to 12 of Table 1); Lanes 16–21: GTPVs (sample 13 to 18 of Table 1), Lanes 22–25: LSDVs (sample 19 to 22 of Table 1)

The optimal assay parameters are presented in Methods. The optimised assay was further evaluated by testing 46 CaPV isolates or clinical samples including 12 field isolates of SPPV, 5 SPP vaccines, 13 field isolates of GTPV and 15 field isolates of LSDV and 1 LSDV vaccine (Table 1). All 5 SPP vaccines produced 218 bp PCR products while the SPPV field isolates produced a 302 bp product (Fig. 2 and Additional file 2: Figure S1). The SPPV field isolates could not be clearly differentiated from GTPVs and LSDVs (Fig. 2 and Additional file 2: Figure S1).

### Limit of detection and specificity of the assay

The limit of detection of the assay was evaluated by amplifying 10-fold serial dilutions of plasmids as described in Methods. The results showed that the limit of detection for SPPV field isolates and vaccine strains were 80 and 10 copies/reaction (Additional file 3: Figure S2) respectively. The specificity of the assay was tested by attempting to amplify non-capripoxvirus DNA from Orf virus, *Mycoplasma capricolum* ssp. *capripneumonia* (Mccp), Bovine herpes virus (BOHV), Bovine popular stomatitis virus (BPSV) and cDNA from peste des petits ruminant virus. No amplification was observed (Additional file 4: Figure S3).

### Sequencing of the PCR amplicons

All PCR amplicons were sequenced for further validation of this PCR approach. The results of the multiple sequence alignment showed the absence of the 84-nucleotide deletion in all SPPV field isolates, GTPV and LSDV. Additionally, the targeted region was found to be well conserved among the SPPV field isolates and the SPPV vaccine strains except for the 84 nucleotides deletion. Ten nucleotide variations were observed between SPPV field isolates and GTPVs. LSDV sequences are longer than SPPV field isolates and GTPVs due to an insertion of 34–36 nucleotides. GTPV Saudi Arabia appeared to be a SPPV and SPPV Oman showed the GTPV specific features. The vaccine KS1 (renamed in our paper as LSDV KS-1) presented a 34-nucleotide insertion as most LSDV of this study.

## Discussion

In this study, we identified a suitable target in CaPV genome and developed a PCR method to discriminate SPP vaccine strains from SPPV field isolates as well as from other CaPVs.

By aligning the full genome sequences of SPPV field isolates with the unpublished full genome of SPPV Morocco vaccine, a Romanian strain, we identified a region of 84 bp nucleotide deletion in the vaccine strains. Primers were designed to amplify this intergenic region located between the DNA ligase gene and the VARV B22R homologue gene of CaPV. The sequencing of this region in SPPV vaccine strains and SPPV field isolates available for this study, showed this deletion to be unique to the SPP vaccines derived from the Romanian and the Yugoslavian RM/65 strains produced in Algeria, Egypt, Morocco and Senegal. However, the sequence of the NISKHI vaccine, whose full genome has been previously published [9] did not carry such a deletion. Nevertheless, the use of the NISHKI strain seems to be confined to Russia and countries of the former Soviet Union such as Kazakhstan [10]. By using the targeted intergenic region, the aim of this study was to develop an assay to discriminate the live attenuated Romanian and Yugoslavian RM/65 SPP vaccines strains from virulent SPPV field isolates and other CaPVs.

Interestingly, Romanian and Yugoslavian RM/65 SPPV vaccines produced amplicons of shorter lengths, as compared to field isolates of SPPV and other CaPVs, and thus could be easily discriminated from them. The assay was found to be very specific and sensitive, and the accuracy and reliability was confirmed by sequencing the corresponding amplicons of SPP vaccine strains and SPPV, GTPV and LSDV field isolates available for this study.

The high sensitivity of this assay for SPPV vaccine strain, as compared to the field isolates, is likely due to the shortest amplicon size in the vaccine strains. No amplification was detected for any non-CaPV samples by the assay. However, the assay could not differentiate SPPV field isolates from LSDVs and GTPVs. Although an insertion of 34–36 nucleotides was observed in all LSDVs, making them longer than GTPVs and SPPV field isolates, it was not possible to resolve these differences on the agarose gel. Owing to the availability of species-specific PCR [11–13] for CaPV genotype determination, we suggest that the current assay could be used once the genotype of CaPV is established. Alternatively, the exact capripoxvirus genotype can be determined by sequencing the region targeted in this study. Within the SPPV genotype, the test, undoubtedly discriminates between the virulent field isolates and the vaccines derived from the Romanian and the Yugoslavian RM/65 strains.

The availability of an easy-to-use molecular method is needed for the identification of SPPV, to rule out the involvement of SPP vaccines following a CaP outbreak in previously vaccinated flock of small ruminants. In Middle Eastern and Asian countries, small ruminants are protected from CaPV infections using various SPPV, GTPV or LSDV derived vaccines, however, SPP vaccines derived from Romanian and the Yugoslavian RM/65 strains are predominant [14]. In Africa, small ruminants are protected against CaPV using either KSGP O-240 and O-180 vaccines or SPP vaccines derived from the Romanian and the Yugoslavian RM/65 strains. Since KSGP O-240 and O-180 strains are of LSDV genotype, the use of a CaPV species-specific assay [11–13] can allow for determination of their involvement when the disease occurs in a previously vaccinated herd. However, if a herd is vaccinated with a SPP vaccine from the Romanian and the Yugoslavian RM/65 strains, the full genome sequencing of the viral isolate, collected during an outbreak, would be required to rule out vaccine involvement when an outbreak occurs. This is time-consuming and cost prohibitive for most laboratories in limited resourced countries. Thus, the identification of suitable target in the viral genome to differentiate SPPV vaccine strains from SPPV field isolates greatly reduces the costs, by allowing the sequencing of small specific genome fragments. Furthermore, this region can be targeted in a simple molecular method such as the PCR approach presented in the work, thereby avoiding the use of sequencing. The current assay is intended to be used by all veterinary laboratories, including those with limited resources. It can also be used as a front-line tool for the direct screening of pathological samples collected during CaPV outbreaks, especially those occurring in previously vaccinated small ruminant populations. A study was recently conducted in Morocco to rule out the involvement of the vaccine strain in 2010 SPP outbreaks using a PCR based approach [15]. When compared to the assay developed by Haegeman and co-workers [15], our method presents the advantage of using only one primer pair to target both viruses, and thus, is much simpler to conduct and interpret. In addition, more vaccine strains and field isolates, from various geographical locations were included in this study, which broadened the scope of the applicability to all countries where SPP vaccines derived from the Romanian or the Yugoslavian RM/65 strains are used.

## Conclusions

The molecular assay described herein is a reliable and rapid method that can easily be implemented for the differentiation of SPP vaccine derived from the Romanian or the Yugoslavian RM/65 strains from virulent SPPV field isolates. The method is applicable as a routine tool for outbreak investigations and disease surveillance in both SPP and GTP enzootic and disease-free countries. It is expected that its adoption by veterinary laboratories in CaPV affected countries, will help facilitate the control and management of CaP disease in small ruminants.

## Additional files

Additional file 1: Table S1. Non-capripoxvirus samples tested for specificity study. (DOCX 16 kb)

Additional file 2: Figure S1. Gel picture of PCR products for the remaining capripoxvirus samples. These sample were tested in this study, but not presented in Fig. 2 of the manuscript. The PCR products of 218 bp, 302 bp and 338 bp represent SPPV vaccine strains, SPPV field isolates/GTPVs, and LSDVs respectively. First row: MM = 50 bp DNA ladder; a = positive control plasmid of the SPPV field isolates; b = positive control plasmid of the SPPV vaccine strain; c = Negative control; Lane 5 to 15 (sample 23 to 33 in Table 1 of the manuscript). Second row: MM = 50 bp; Lane 2 to 14 (sample 34 to 46 in Table 1 of the manuscript). (PDF 104 kb)

Additional file 3: Figure S2. Determination of the limits of detection of the PCR assay. Defined amount for the plasmid genotype standard (10^4^, 10^3^, 100, 80, 60, 40, 20, 10, 1 and 0) for SPPV vaccine (A) and SPPV field isolates (B) were tested in parallel reactions and run on agarose gel. (PDF 77 kb)

Additional file 4: Figure S3. Gel picture of PCR for the non-capripoxvirus samples tested in this study. MM = 50 bp DNA ladder; a = positive control plasmid of the SPPV field isolates; b = positive control plasmid of the SPPV vaccine strain; c = Negative control; 1–5 (ORF viruses); 6 (BPSV); 7–8 (Mccp); 9 (cDNA, PPRV); 10 (BOHV-1); 11 (BOHV-2). (PDF 45 kb)
