# Supplementary material for: A gel-based PCR method to differentiate sheeppox virus field isolates from vaccine strains
Source: Virol J. 2018 Apr 2;15:59. doi: 10.1186/s12985-018-0969-8 (PMC5879731; doi:10.1186/s12985-018-0969-8)
Supplement: Supplementary file 1 — Table S1. Non-capripoxvirus samples tested for specificity study. (DOCX 16 kb) [file 12985_2018_969_MOESM1_ESM.docx]

Supplement Table 1: Non-capripoxvirus samples tested for specificity study.

|  |  |  | **Sample No.** |  | **Isolate/Strain name** | **Source** | **Sample type** | **Country** | **Host** |
| --- | --- | --- | --- | --- | --- | --- | --- | --- | --- |
|  |  |  | 1 |  | ORFV MB38/13 C-2 | NVI/Ethiopia | Skin scraping | Ethiopia | Sheep |
|  |  |  | 2 |  | ORFV MB38/13 C-3 | NVI/Ethiopia | Skin scraping | Ethiopia | Sheep |
|  |  |  | 3 |  | ORFV MB38/13 C-4 | NVI/Ethiopia | Skin scraping | Ethiopia | Sheep |
|  |  |  | 4 |  | ORFV MB38/13 C-5 | NVI/Ethiopia | Skin scraping | Ethiopia | Goat |
|  |  |  | 5 |  | ORFV MB38/13 C-6 | NVI/Ethiopia | Skin scraping | Ethiopia | Goat |
|  |  |  | 6 |  | BPSV Stamm M1 | HSL-AGES/Austria | Cell culture | Germany | Cattle |
|  |  |  | 7 |  | Mccp | NVI/Ethiopia | Pathological lesions | Ethiopia | Goat |
|  |  |  | 8 |  | Mccp | NVI/Ethiopia | Pathological lesions | Ethiopia | Goat |
|  |  |  | 9 |  | PPRV (cDNA) | NVI/Ethiopia | Nasal swab | Ethiopia | Goat |
|  |  |  | 10 |  | BOHV-1 404/2018 | HSL-AGES/Austria | Cell culture | Austria | Cattle |
|  |  |  | 11 |  | BOHV-2 95-5/2016 | HSL-AGES/Austria | Cell culture | Austria | Cattle |

BOHV = Bovine herpes virus; BPSV = Bovine papular stomatitis virus; Mccp = *Mycoplasma capricolum* ssp. *Capripneumonia*; NVI = National Veterinary Institute; HSL-AGES = High Security Laboratory, Austrian Agency for Health and Food Safety; CVRL = Central Veterinary Research Laboratories
