# Supplementary figures and images for: A gel-based PCR method to differentiate sheeppox virus field isolates from vaccine strains
Source: Virol J. 2018 Apr 2;15:59. doi: 10.1186/s12985-018-0969-8 (PMC5879731; doi:10.1186/s12985-018-0969-8)

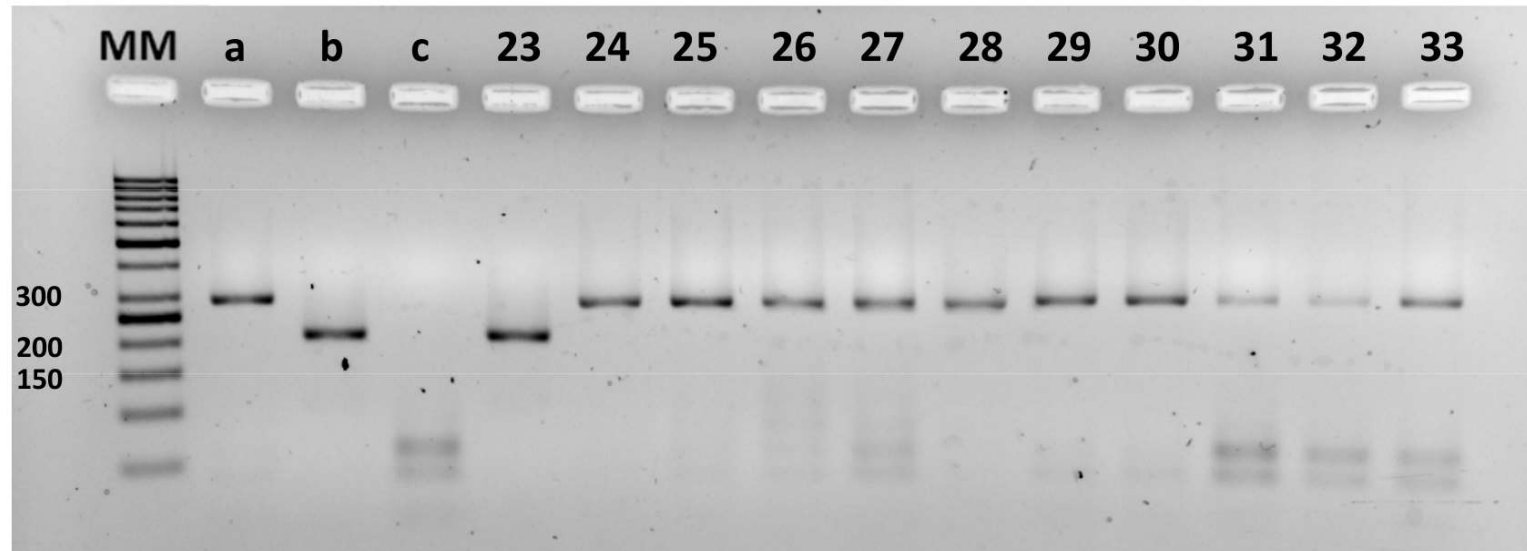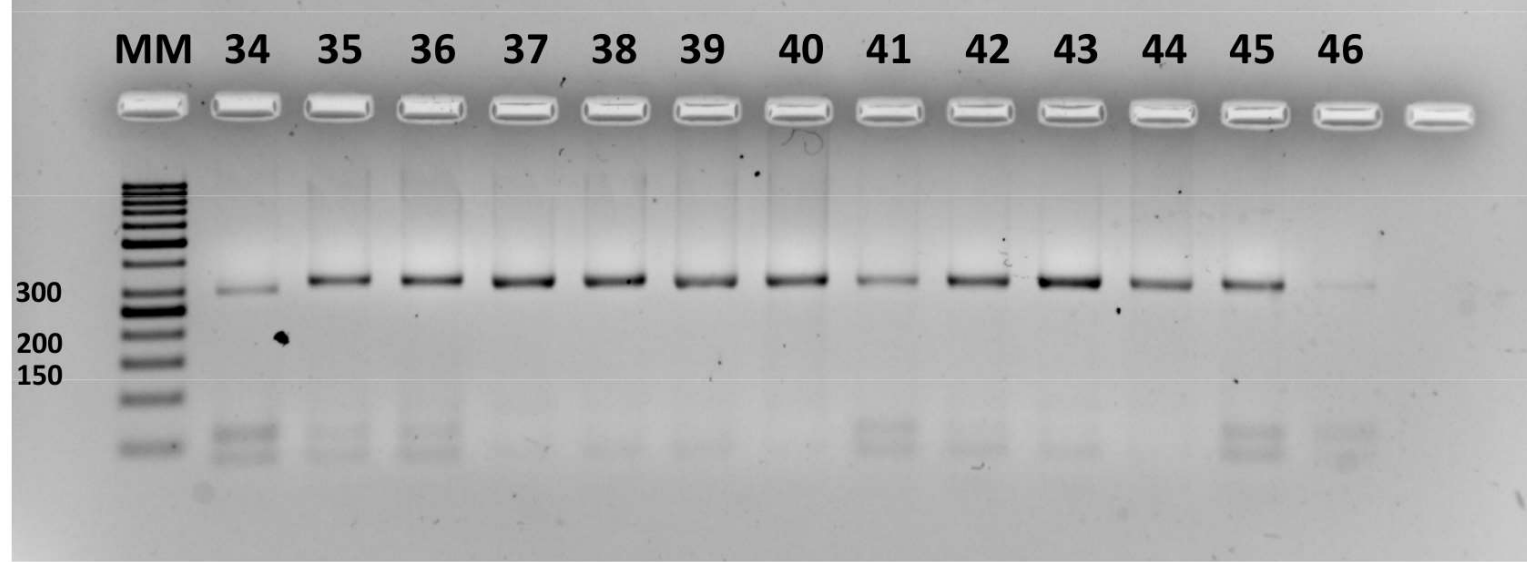

Supplement: Supplementary file 2 — Figure S1. Gel picture of PCR products for the remaining capripoxvirus samples. These sample were tested in this study, but not presented in Fig. 2 of the manuscript. The PCR products of 218 bp, 302 bp and 338 bp represent SPPV vaccine strains, SPPV field isolates/GTPVs, and LSDVs respectively. First row: MM = 50 bp DNA ladder; a = positive control plasmid of the SPPV field isolates; b = positive control plasmid of the SPPV vaccine strain; c = Negative control; Lane 5 to 15 (sample 23 to 33 in Table 1 of the manuscript). Second row: MM = 50 bp; Lane 2 to 14 (sample 34 to 46 in Table 1 of the manuscript). (PDF 104 kb) [file 12985_2018_969_MOESM2_ESM.pdf]

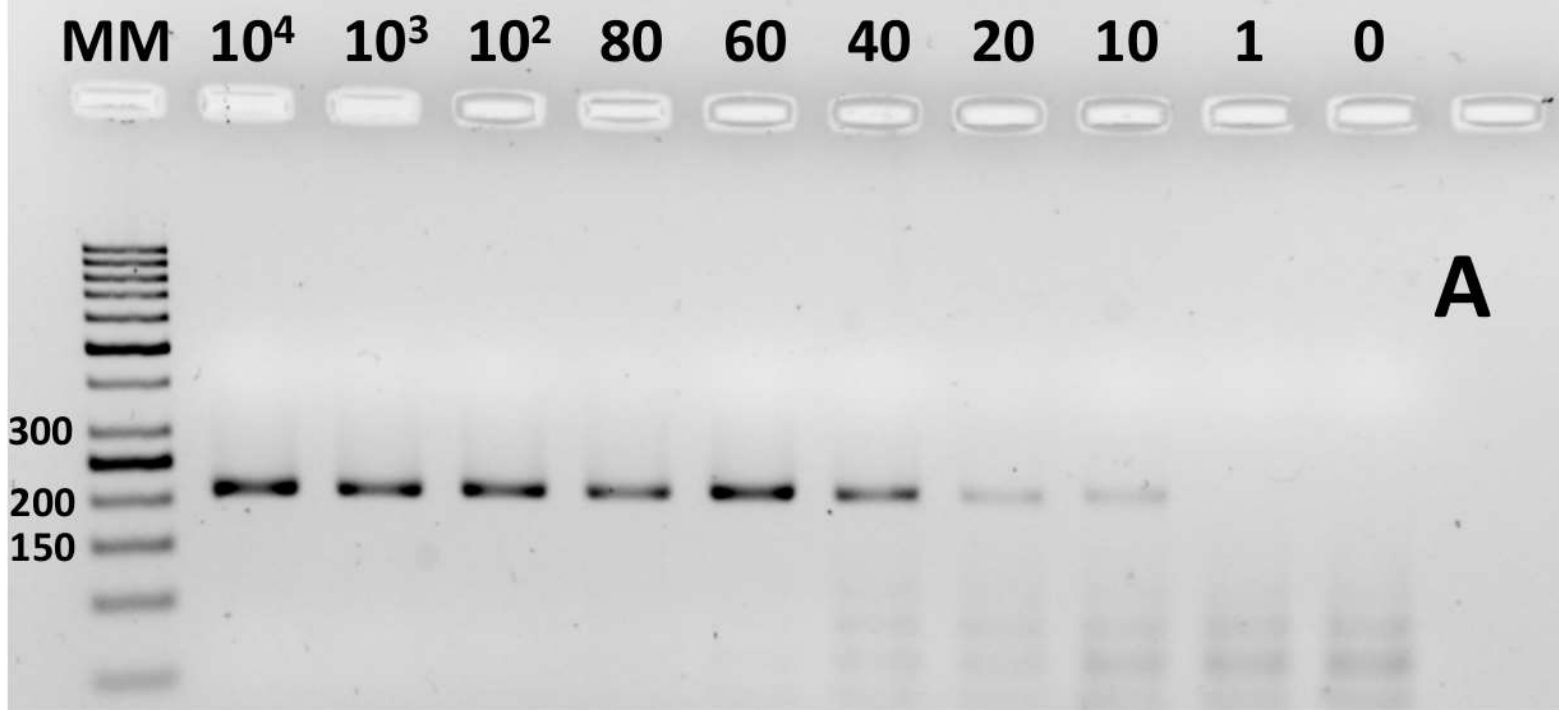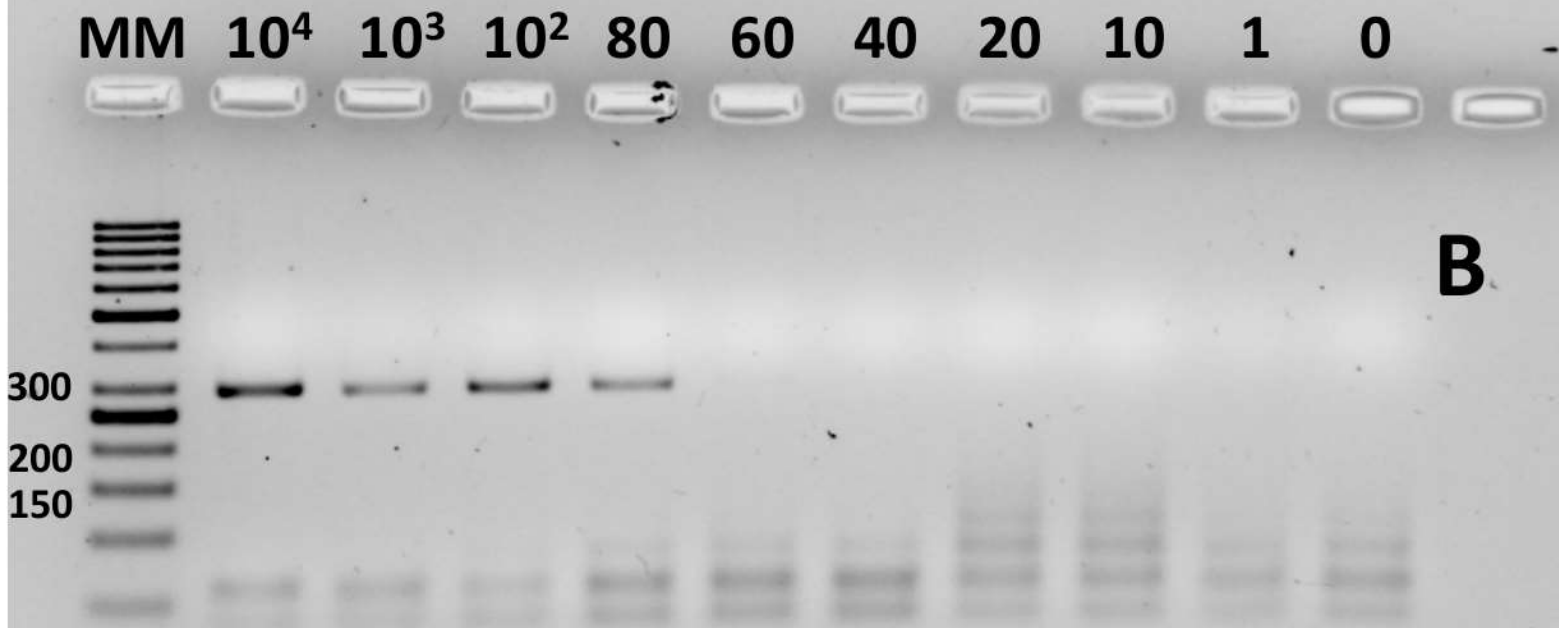

Supplement: Supplementary file 3 — Figure S2. Determination of the limits of detection of the PCR assay. Defined amount for the plasmid genotype standard (104, 103, 100, 80, 60, 40, 20, 10, 1 and 0) for SPPV vaccine (A) and SPPV field isolates (B) were tested in parallel reactions and run on agarose gel. (PDF 77 kb) [file 12985_2018_969_MOESM3_ESM.pdf]

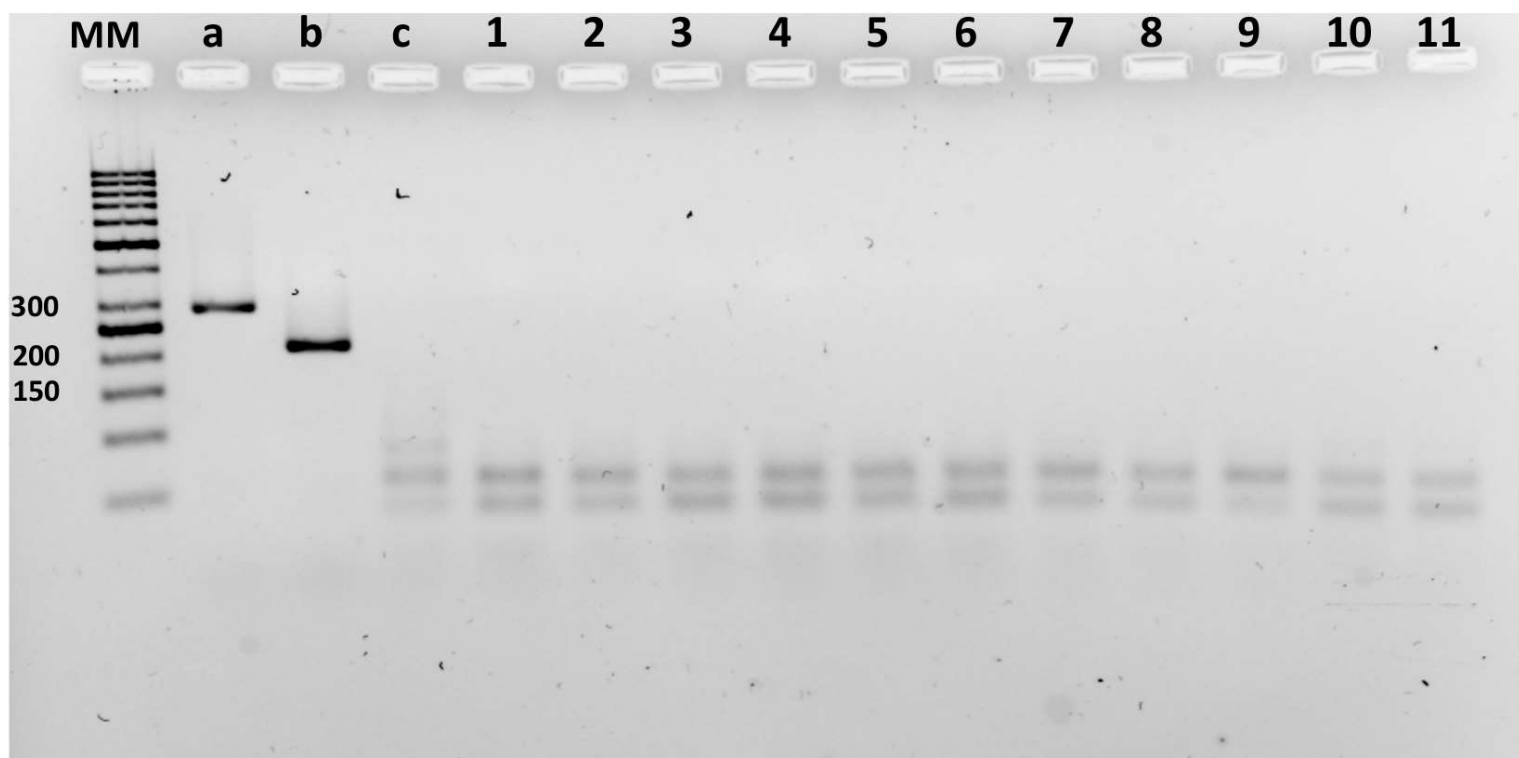

Supplement: Supplementary file 4 — Figure S3. Gel picture of PCR for the non-capripoxvirus samples tested in this study. MM = 50 bp DNA ladder; a = positive control plasmid of the SPPV field isolates; b = positive control plasmid of the SPPV vaccine strain; c = Negative control; 1–5 (ORF viruses); 6 (BPSV); 7–8 (Mccp); 9 (cDNA, PPRV); 10 (BOHV-1); 11 (BOHV-2). (PDF 45 kb) [file 12985_2018_969_MOESM4_ESM.pdf]
